# Beetles (Coleoptera) in deciduous dead wood tree species trunks in Lithuania

**DOI:** 10.3897/BDJ.11.e106132

**Published:** 2023-07-04

**Authors:** Aistė Lekoveckaitė, Virginija Podėnienė, Romas Ferenca

**Affiliations:** 1 Vilnius University, Life Sciences Center, Vilnius, Lithuania Vilnius University, Life Sciences Center Vilnius Lithuania; 2 Kaunas T. Ivanauskas Zoological Museum, Kaunas, Lithuania Kaunas T. Ivanauskas Zoological Museum Kaunas Lithuania

**Keywords:** saproxylic, trunk-emergence traps, wind-felled trees

## Abstract

We present a list of beetles that emerged from wind-felled tree trunks of several tree species, including European ash (*Fraxinusexcelsior*), aspen (*Populustremula*), common oak (*Quercusrobur*), birch (*Betula* sp.), small-leaved linden (*Tiliacordata*) and black alder (*Alnusglutinosa*). Four hundred and ninety species and 60 families of beetles were collected using trunk-emergence type traps. We found 440 beetle species that had previously been recorded from dead wood; the remaining 50 were newly discovered and all were considered as not directly associated with dead wood. Common oak trunks had the highest diversity of beetles, with approximately 42% of the identified beetle species found in our research. Of all the beetle species identified in the study, about half are saproxylic, while the remaining are considered as not having direct association with dead wood. The results of the study emphasise the importance of dead wood in maintaining beetle diversity in Lithuanian forests. This study provides a valuable baseline for future research on beetles in dead wood in Lithuania and may help to provide information for conservation efforts to protect these important habitats.

## Introduction

Forest land occupies 33.8% of Lithuania (Government of the Republic of Lithuania 2022). Lithuanian forests belong to the European hemi-boreal mixed broadleaved-coniferous forest type and around 44% of the country's forests consist of deciduous tree species (Varnagirytė-Kabašinskienė et al. 2019). Amongst them, *Betula* sp. occupies 22.2% of the forest area, *Populustremula* stands occupy 4.6%, *Alnusglutinosa* and *Alnusincana* occupy 7.6% and 5.9%, respectively, *Quercusrobur* and *Fraxinusexcelsior* occupy 2.2% and 0.9%, respectively, while areas covered by other deciduous tree species are insignificant (1.1%) (Varnagirytė-Kabašinskienė et al. 2019). According to the recent state accounting of Lithuanian forests, which summarises information about forest resources, their quality, natural and economic condition, the trends in the species composition of stands have changed little over the past few years (Government of the Republic of Lithuania 2022). However, areas of conifer, black alder and oak stands are increasing, while areas covered by birch, aspen, white alder and ash are shrinking (Government of the Republic of Lithuania 2022).

In a healthy forest, wood comes in many forms, including living trees, stumps, snags, logs and branches (Paletto and Tosi 2010). These forms of wood host a variety of species, mainly including fungi and insects (Stokland et al. 2012, Jacobsen et al. 2015). One of the most important group of insects are saproxylic beetles, which depend on dead or dying wood for some part of their life cycle (Speight 1989). Beetles play an important role in decomposing and recycling dead wood. In fact, more than half of forest-dwelling beetles may be saproxylic (Bouget et al. 2008). Beetles can be grouped, based on their lifestyle – obligate or facultative saproxyls (e.g. Maňák and Schlaghamerský (2009), Milberg et al. (2014), Milberg et al. (2016)), some are flexible and can live on a variety of dead tree species, while others are specific to one type of host tree (Jonsell et al. 1998, Abrahamsson et al. 2009, Toivanen and Kotiaho 2010, Milberg et al. 2014).

Numerous studies have been conducted to investigate various aspects of beetle assemblages, including their relationships to environmental conditions and associations with different tree species (Horák 2011, Macagno et al. 2015, Muñoz-López et al. 2016, Procházka and Schlaghamerský 2019, Zuo et al. 2021, Edelmann et al. 2022). Tree species identity has been identified as one of the most significant drivers determining the community composition of beetles (Müller et al. 2020). While the number of species showing strict host-specificity is relatively low (Jonsson et al. 2005), beetle communities, in general, are dependent on tree species and this dependence decreases significantly as decay progresses (Wende et al. 2017, Zuo et al. 2021). According to research in north Europe (Jonsson et al. 2005), out of nearly 7000 wood-living species in Sweden, only around 130 have been found to exclusively inhabit a single tree species. Additionally, birch, oak and aspen are amongst the most species-rich deciduous trees in the country. Taking south-eastern Sweden as an example, 19 out of 171 beetle species had a significant association with common oak, six with Norway maple, two with European ash and five with small-leaved linden (Milberg et al. 2014). Association between beetle species and oaks was also found by investigating hollow oaks, which are rich in dead branches (Sverdrup-Thygeson et al. 2010). The study identified 62 Red-listed beetle species associated with oaks, including 23 oak specialists amongst the 62 oak-associated beetles. These oak specialists belonged to various families, such as Ptiliidae, Leiodidae, Scydmaenidae, Staphylinidae, Scarabaeidae, Elateridae, Cantharidae, Anobiidae, Lymexylidae, Melyridae, Nitidulidae, Tenebrionidae, Aderidae and Scraptiidae. A study in Germany revealed the host preference of saproxylic beetle communities on logs of 13 tree species, including birch, European ash, aspen, oak and linden, over a period of two years after harvesting (Müller et al. 2015). The analysis of 381 saproxylic beetle species that emerged from the logs showed that European hornbeam (*Carpinusbetulus*) was the most preferred tree species, while European ash, Douglas-fir (*Pseudotsugamenziesii*), European larch (*Larixdecidua*) and linden (*Tilia* sp.) were the least preferred.

However, it is not always easy to define whether a species living in deadwood depends on wood fibres, fungal hyphae or other factors (Bakke 1999) and identify beetle-tree associations. Despite that, further studies on the diversity of saproxylic beetles in different tree species and countries are necessary to apply the findings to the conservation and enrichment of unique deadwood habitats and their associated beetles.

More than 3600 species of beetles are recorded in Lithuania up to date (Tamutis et al. 2011, Ferenca et al. 2011, Nagrockaitė et al. 2011, Tamutis 2012, Ferenca et al. 2013, Ivinskis et al. 2013, Monsevičius 2013, Ivinskis et al. 2014, Tamutis and Barševskis 2014, Ferenca and Tamutis 2015, Ivinskis et al. 2015, Tamutis et al. 2015, Ferenca et al. 2016, Paukkunen et al. 2016, Ivinskis et al. 2017, Lekoveckaitė et al. 2017, Pacevičius 2017, Ferenca et al. 2018, Lekoveckaitė et al. 2019, Tamutis and Martinaitis 2019, Monsevičius 2020, Monsevičius 2022). Very little research on beetles in Lithuania has been related to dead wood (Ferenca and Tamutis 2011, Ivinskis et al. 2017, Lekoveckaitė et al. 2017, Pacevičius 2017, Ferenca et al. 2018, Lekoveckaitė et al. 2019, Monsevičius 2020). Our study is the first extended research, designed to investigate beetle communities in deciduous tree species dead wood and especially in its early stage of decay.

## Material and methods

**Figure 1.**
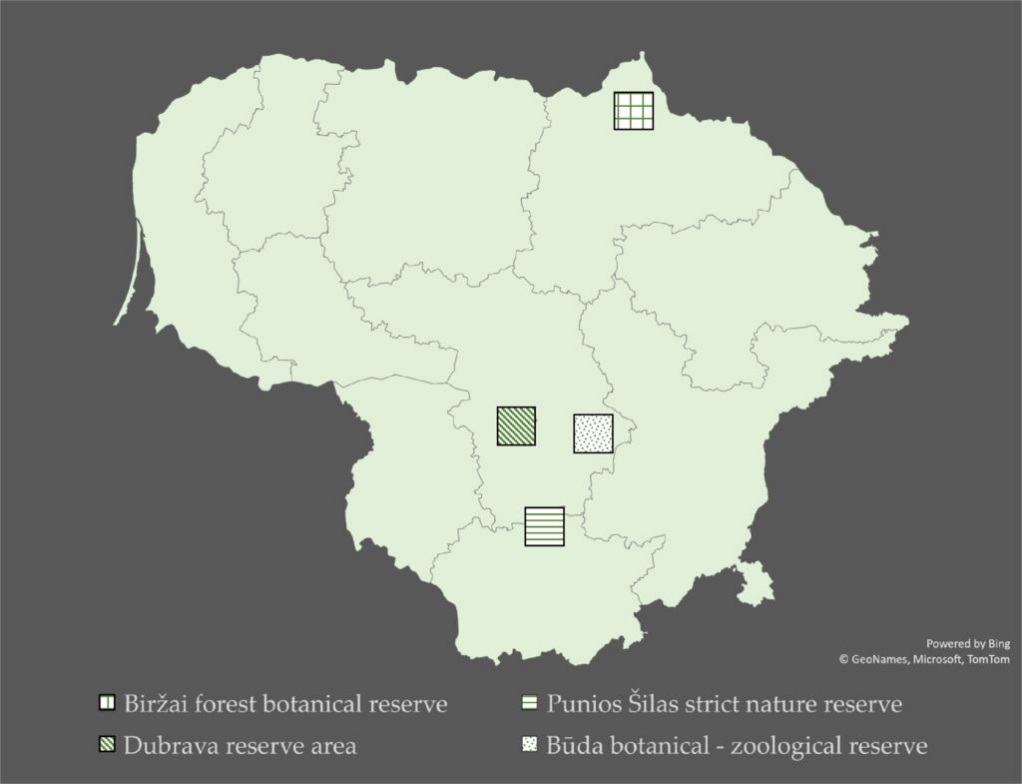
Map of Lithuania with marked research locations.

From 2018 to 2021, we collected saproxylic beetle fauna in four protected forest areas of Lithuania (Table 1, Fig. 1). The chosen forests are part of the Natura 2000 network where main forest felling is prohibited or limited to low-intensity selective felling, sanitary felling is also restricted and additional uncut trees must be left in clearings. Additionally, dry trees cannot be felled. Dead wood in the studied forests mainly includes dead branches, standing dead trees (snags) and trees felled by the wind.

A total of 54 deciduous wind-felled trees belonging to six different tree species were chosen for the research (Table 1). Tree species were identified, considering the bark of the tree and the general composition of the stand. As beetle species associated with a specific tree species decrease with increasing decomposition degree, we assumed that primary decay stages should host high beetle diversity. Instead of selecting recently deceased trees in the first stage of decay, we chose weakly-decayed trees in the second stage. The second stage of wood decay was identified, based on loose bark and knife blade penetration of less than 2 cm (Renvall 1995, Palviainen et al. 2008). The diameters of the tree varied from 21 to 53 cm.

**Figure 2.**
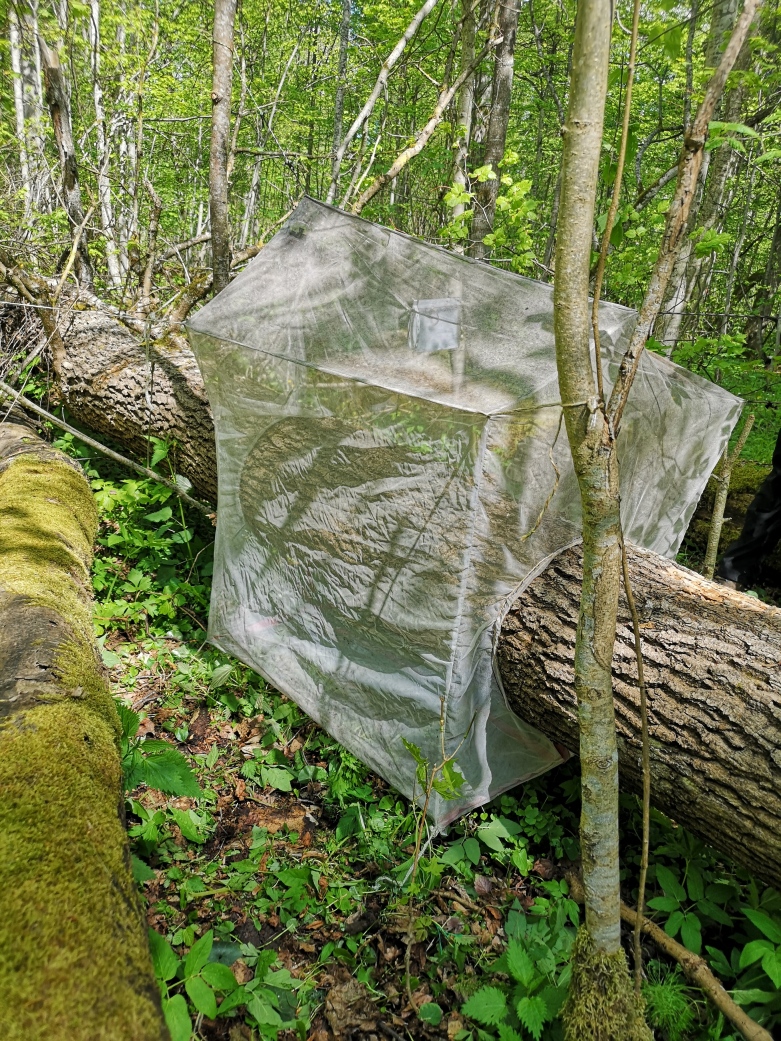
Modified trunk-emergence trap.

We used a modified trunk-emergence trap model to collect beetle specimens (Halme et al. 2013) (Fig. 2). Traps were sewn from transparent, air-permeable polyester cloth, to maintain the microclimatic conditions inside the trap unaltered. All traps were designed to cover a 1-metre section of the wind-felled tree and one vertical wall of the trap is longer than the other to make the insects emerging from the wood fly to the highest point. The bottom of each trap was sealed by joining the cloth with contact tape. For the traps to be properly installed, the trunk must be raised off the ground. We installed traps on the middle parts of such trunks, stretching their walls with the help of sewn ropes so that the material does not form wrinkles. A two-piece collecting jar was attached to the highest point of the trap and filled with > 99% propylene glycol and emptied every two weeks from June to October. In total, we took 82 samples during the four years.

Beetle specimens were identified at the species level. The species names were used following De Jong et al. (2014). The collected material is deposited in the Tadas Ivanauskas Kaunas Zoological Museum and the Vilnius University Life Sciences Center Museum of Zoology.

## Results

A total of 6796 coleopteran specimens belonging to 60 families and 490 species were collected in 54 studied tree trunks of the second stage of wood decay. A small number of collected specimens were identified to the genus level and regarded as separate species (due to the morphology of specimens) (Table 2).

**Figure 3.**
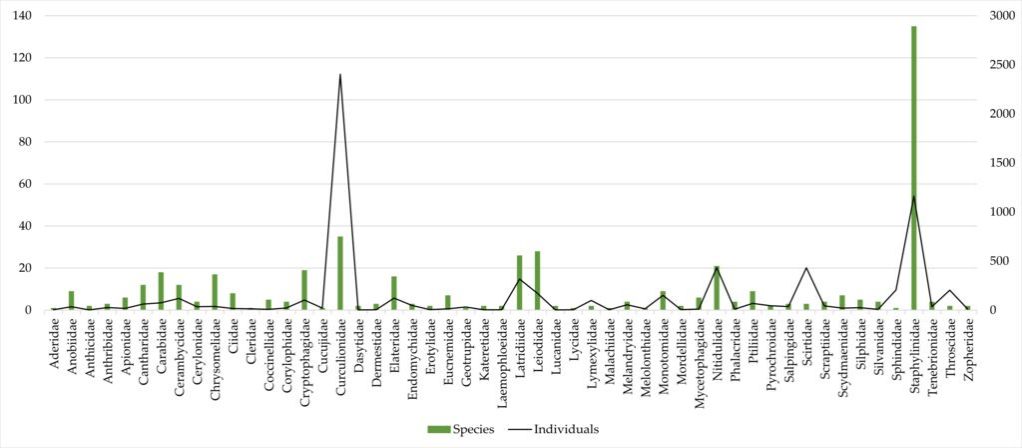
Number of individuals (line graphs) and species (bar graphs) of beetle families collected during the research. Families of beetles, which had one species and one individual each, were not included in the graph (Biphyllidae, Byrrhidae, Histeridae, Hydraenidae, Lampyridae, Nemonychidae, Tetratomidae, Trogositidae).

The most diverse beetle families were Staphylinidae (135 species), Curculionidae (35 species), Leiodidae (27 species) and Latridiidae (26 species) (Fig. 3, Table 2). Together, they represent 45.51% of collected species in dead wood trunks. Sixteen beetle families were represented by a single species each (Aderidae, Biphyllidae, Byrrhidae, Cleridae, Cucujidae, Geotrupidae, Histeridae, Hydraenidae, Lampyridae, Lycidae, Malachiidae, Melolonthidae, Nemonychidae, Sphindidae, Tetratomidae, Trogositidae) (Fig. 3, Table 2). Other beetle families contained from 2 to 21 species (Fig. 3, Table 2).

Curculionidae (2403 specimens) and Staphylinidae (1162 specimens) were the most abundant families (Fig. 3, Table 2). Together they represent 52.46% of collected specimens. Eight beetle families (Biphyllidae, Byrrhidae, Histeridae, Hydraenidae, Lampyridae, Nemonychidae, Tetratomidae and Trogositidae) (Fig. 3, Table 2) were represented only by a single specimen, while the majority of beetle families contained from 2 to 433 specimens.

Amongst the species, the most abundant were *Trypodendronsignatum* (Fabricius, 1787) (1225 specimens), *Cyphonochraceus* Stephens, 1830 (408 specimens), *Xyleborusdispar* (Fabricius, 1792) (313 specimens), *Dryocoetesautographus* (Ratzeburg, 1837) (223 specimens), *Hylesinuscrenatus* (Fabricius, 1787) (212 specimens), *Aspidiphorusorbiculatus* (Gyllenhal, 1808) (204 specimens), *Trixagusdermestoides* (Linnaeus, 1766) (199 specimens), *Glischrochilushortensis* (Geoffroy in Fourcroy, 1785) (168 specimens) and *Bolitocharaobliqua* Erichson, 1837 (142 specimens). Other beetle species abundance varied from 1 to 98 specimens (Table 2).

**Figure 4.**
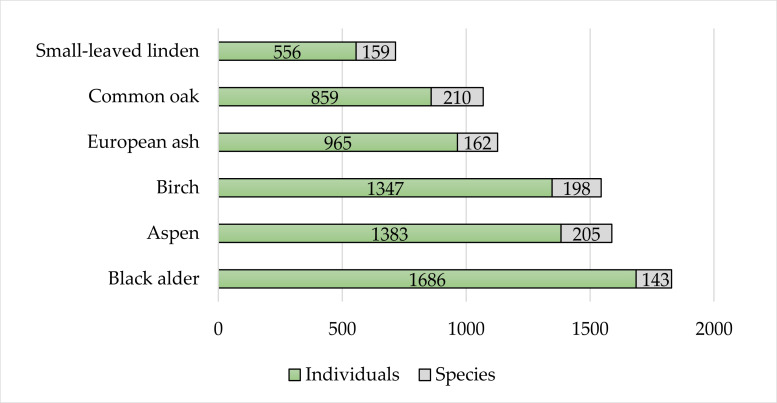
Richness and abundance of beetles in six different tree species. Note: the number of decaying tree trunks sampled with emergence traps was not equal amongst all six tree species. There were 12 trunks sampled from European ash, six from small-leaved linden and nine from each of the remaining species.

In total, 965 specimens belonging to 162 beetle species emerged from trunks of European ash, 1383 specimens and 205 species – from trunks of aspen, 1686 specimens and 143 species – from trunks of Black alder, 1347 specimens and 198 species – from trunks of birch, 556 specimens and 159 species – from trunks of small-leaved linden and 859 specimens and 210 species – from trunks of common oak (Fig. 4, Table 2).

**Figure 5.**
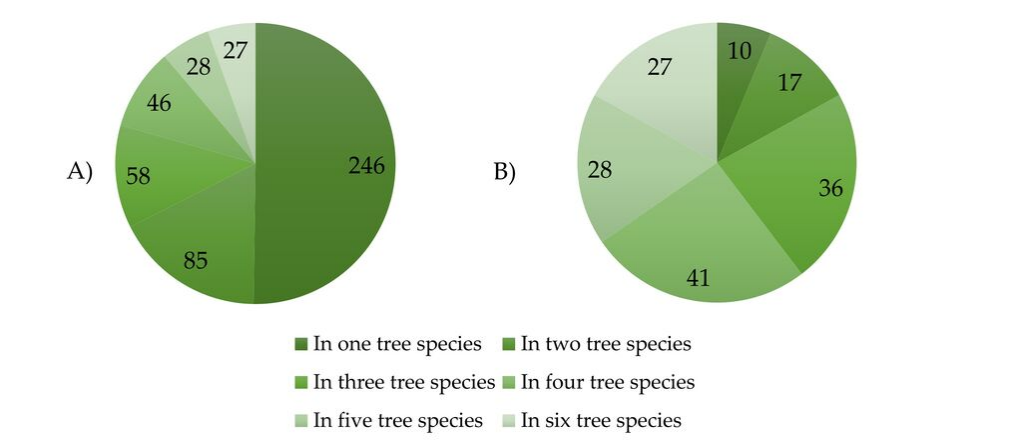
The number of beetle species found in one or more tree species, including: A) the total number of beetle species and B) the number of beetle species after excluding those with five or fewer collected specimens.

Out of the 490 beetle species collected in the research, almost half (246) were found in only one tree species. A total of 85 species were found in two tree species, 58 in three tree species, 46 in four tree species, 28 in five tree species and 27 in all six tree species (Fig. 5). Excluding beetle species with five or fewer specimens, there were 10 beetle species in one tree species, 17 in two tree species, 36 in three tree species, 41 in four tree species, 28 in five tree species and 27 in six tree species (Fig. 5).

## Discussion

Our research is the first thorough study of saproxylic beetles using the emergence type of traps in Lithuania. It has revealed a huge diversity of saproxylic beetles, accounting for about 13% of all known beetle species in Lithuania. A range of methods is used to collect saproxylic beetles (Ranius and Jansson 2002, Jonsell and Hansson 2007, Peuhu et al. 2019), with window traps, trunk window traps and emergence or eclector traps amongst the most popular (Peuhu et al. 2019). A similar method of closed emergence traps for collecting beetles has been used in Germany (Irmler et al. 1996, Müller et al. 2015), France (Bouget et al. 2012), Italy (Parisi et al. 2021) and Sweden (Wikars et al. 2005, Gibb et al. 2006, Hjältén et al. 2010). For example, in Germany, with half the survey time, but with considerably more trunks and almost twice as many tree species, a lower beetle diversity (381 species) was collected compared to our study (Müller et al. 2015).

Of the 490 beetle species we identified, 440 were previously discovered in dead wood of various tree species: European ash, small-leaved linden, common oak, aspen, birch, black alder, Norway spruce (*Piceaabies*), pine (*Pinussylvestris*), common beech (*Fagussylvatica*), silver fir (*Abiesalba*), rowan (*Sorbusaucuparia*) and goat willow (*Salixcaprea*), which are the most common tree species in Europe (Kaila 1993, Irmler et al. 1996, Martikainen 2001, Jonsell et al. 2004, Lindhe and Lindelöw 2004, Wikars et al. 2005, Byk et al. 2006, Gibb et al. 2006, Müller et al. 2007, Djupström et al. 2008, Müller and Bussler 2008, Unal et al. 2009, Hjältén et al. 2010, Horák 2011, Ranius et al. 2011, Bouget et al. 2012, Jonsell 2012, Lassauce et al. 2013, Sawoniewicz 2013, Vindstad et al. 2014, Redolfi De Zan et al. 2014, Papis and Mokrzycki 2015, Milberg et al. 2016, Seibold et al. 2016, Selberg 2019, Procházka and Schlaghamerský 2019, Parisi et al. 2021, Vogel et al. 2021, Mazur et al. 2021, Graf et al. 2022). Out of the 198 beetle species identified from birch dead wood, about a half (91 species) were also presented in beetle species lists obtained from Germany (Vogel et al. 2021), Poland (Sawoniewicz 2013), Norway (Kaila 1993, Vindstad et al. 2014) and Sweden (Lindhe and Lindelöw 2004). Amongst 210 beetle species collected from common oak deadwood, a total of 116 overlapping with beetle species lists were obtained in oaks in Germany (Vogel et al. 2021), France (Bouget et al. 2012, Lassauce et al. 2013) and Sweden (Lindhe and Lindelöw 2004, Milberg et al. 2016). Fifty-five beetle species collected from dead aspen overlapped when compared with research conducted in Germany (Vogel et al. 2021), Finland (Martikainen 2001, Ranius et al. 2011) and Sweden (Lindhe and Lindelöw 2004, Selberg 2019). We did not find many listings of beetles in dead wood of small-leaved linden. However, 48 beetle species collected in our research overlap with species obtained in several works (Jonsell 2012, Vogel et al. 2021). Limited research and species lists make it difficult to compare our findings on beetles in dead wood of European ashes and black alders. However, comparing our study with research in Germany, we found overlapping beetle species in both trees: three in European ashes and four in black alders (Vogel et al. 2021). Although the number of overlapping species is relatively small, this suggests that the beetle species in dead European ash and black alder may be much more similar than they appear to be at present, but further research on the dead wood of these tree species is needed. The comparison of beetle lists and other researchers' findings highlights the significance of tree species of beetle communities (Zuo et al. 2021).

Based on other authors' species lists and species biology, beetle species regarded as saproxylic (according to Speight (1989)) accounted for about a half of our collected beetles, while the remaining are considered as non-wood dependent species (Hågvar and Økland 1997, Schmidl and Bußler 2004, Wikars et al. 2005, Byk et al. 2006, Gibb et al. 2006, Brunet and Isacsson 2008, Maňák and Schlaghamerský 2009, Horák 2011, De Biase 2011, Vodka and Cizek 2013, Sawoniewicz 2013, Milberg et al. 2014, Vindstad et al. 2014, Anonymous 2015, Papis and Mokrzycki 2015, Carlsson et al. 2016, Seibold et al. 2016, Procházka and Schlaghamerský 2019, Marker 2019, Ekström 2020). Our research, as well as that of other scientists, includes a range of beetle species whose biology is not directly related to this unique habitat and they can be considered incidental to dead wood. Research from neighbouring Poland reveals (Anonymous 2015) that the most of the non-wood-dependent beetles we have collected are associated with accumulated organic matter, plant litter, mud and soil, various plants, mosses, the nests of birds and other animals, carrion and fungal fruiting bodies (Table 2). Staphylinidae was one of the most diverse families in our research. About half of the rove beetles collected are not associated with dead wood and were characterised by relatively low abundance (Table 2).

The high number of non-saproxylic as well as saproxylic beetle species that we found associated with dead deciduous tree species in Lithuania highlights the importance of dead wood for conservation of the overall forest beetle community.

## Figures and Tables

**Table 1.** Study sites in four different years with six species of tree trunks. The brackets indicate the number of traps used.

	**Biržai forest botanical reserve**	**Būda botanical - zoological reserve**	**Dubrava reserve area**	**Punios Šilas strict nature reserve**
**2018**	*F.excelsior* (3) *A.glutinosa* (3)	*F.excelsior* (3) *P.tremula* (3)		
**2019**	*F.excelsior* (3) *A.glutinosa* (3)	*F.excelsior* (3) *P.tremula* (3)		
**2020**		*Q.robur* (3) *T.cordata* (3)		*Q.robur* (3) *T.cordata* (3)
**2021**		*A.glutinosa* (3) *Betula* sp. (3)	*P.tremula* (3) *Betula* sp. (3)	*Q.robur* (3) *Betula* sp. (3)

**Table 2.** List of beetle families and species and their abundance collected in emergence traps fixed to horizontal trunks of six different tree species in the secondary stage of decay in Lithuania, June to October, 2018 to 2021: F. ex. – *Fraxinusexcelsior*, P. tr. – *Populustremula*, A. gl. – *Alnusglutinosa*, B. sp. – *Betula* sp., T. co. – *Tiliacordata*, Q. ro. – *Quercusrobur*.

**Family**	**Species**	**F. ex**	**P.** **tr**	**A.** **gl**	**B. sp.**	**T. co**	**Q. ro**
Aderidae	^1^*Phytobaenusamabilis* Sahlberg, 1834		1		3		
Anobiidae	^1^*Anobiumpunctatum* (De Geer, 1774)			1			
	^1^Dorcatoma (Dorcatoma) dresdensis Herbst, 1792		1				
	^1^Dorcatoma (Pilosodorcatoma) chrysomelina Sturm, 1837				2	1	1
	^1^Ptinus (Bruchoptinus) rufipes Olivier, 1790						3
	^2^Ptinus (Gynopterus) dubius Sturm, 1837						1
	^2^Ptinus (Ptinus) clavipes Panzer, 1806				1	1	
	^1^Ptinus (Ptinus) fur (Linnaeus, 1758)						1
	^2^Ptinus (Ptinus) pilosus Muller, 1821	1					
	^1^Ptinus (Ptinus) subpilosus Sturm, 1837				2	6	12
Anthicidae	^2^*Notoxusmonoceros* (Linnaeus, 1760)					1	
	^2^*Omonadusfloralis* (Linnaeus, 1758)				1		
Anthribidae	^1^*Anthribusnebulosus* Forster, 1770		1		3	1	
	^1^*Dissoleucasniveirostris* (Fabricius, 1798)						2
	^1^*Platystomosalbinus* (Linnaeus, 1758)	11	3	1	2	1	1
Apionidae	^3^*Betulapionsimile* (Kirby, 1811)		2		1	1	2
	^3^*Catapionpubescens* (Kirby, 1811)		2				
	^3^*Catapionseniculus* (Kirby, 1808)	3	1			2	1
	^3^*Kalcapionpallipes* (Kirby, 1808)			1			
	^3^*Oxystomacraccae* (Linnaeus, 1767)		1				
	^3^*Taeniapionurticarium* (Herbst, 1784)				1		
Biphyllidae	^1^*Diplocoelusfagi* Guérin-Méneville, 1838		1				
Byrrhidae	^4^Byrrhus (Byrrhus) pilula (Linnaeus, 1758)						1
Cantharidae	^2^Cantharis (Cantharis) nigricans Muller, 1766	1					2
	^1^*Malthinusfacialis* Thomson, 1864				1		
	^1^*Malthinusflaveolus* (Herbst, 1786)	3			3		
	^1^Malthodes (Malthodes) crassicornis (Mäklin, 1846)					1	1
	^1^Malthodes (Malthodes) fuscus (Waltl, 1838)	2					
	^1^Malthodes (Malthodes) guttifer Kiesenwetter, 1852			4	4		1
	^1^Malthodes (Malthodes) marginatus (Latreille, 1806)	1		1	4	2	2
	^1^Malthodes (Malthodes) minimus (Linnaeus, 1758)	3		1	1		1
	*Malthodes* sp. Kiesenwetter, 1852	5	3	3			
	^2^Podistra (Absidia) rufotestacea (Letzner, 1845)	1					
	^2^Rhagonycha (Rhagonycha) fulva (Scopoli, 1763)				1		1
	^2^Rhagonycha (Rhagonycha) testacea (Linnaeus, 1758)	2	4	1			1
Carabidae	^2^Agonum (Europhilus) thoreyi Dejean, 1828		1				
	^2^Amara (Amara) aenea (De Geer, 1774)		1				
	^2^Amara (Amara) familiaris (Duftschmid, 1812)					3	1
	^2^Calathus (Amphyginus) rotundicollis Dejean, 1828						1
	^2^Carabus (Carabus) granulatus Linnaeus, 1758	3	6	5	9	1	1
	^2^*Cychruscaraboides* (Linnaeus, 1758)		1				
	^2^Dromius (Dromius) quadraticollis Morawitz, 1862					1	
	^1^Dyschiriodes (Eudyschirius) globosus (Herbst, 1783)			1	2		
	^2^Leistus (Leistus) piceus Frölich, 1799	6			1		
	^2^Leistus (Leistus) terminatus (Panzer, 1793)			1	2		1
	^1^*Limodromusassimilis* (Paykull, 1790)		3	1	1	1	
	^2^*Loricerapilicornis* (Fabricius, 1775)						1
	^2^Nebria (Nebria) brevicollis (Fabricius, 1792)					2	
	^1^Pterostichus (Bothriopterus) oblongopunctatus (Fabricius, 1787)		1		3		2
	^2^Pterostichus (Eosteropus) aethiops (Panzer, 1796)			1			
	^2^Pterostichus (Morphnosoma) melanarius (Illiger, 1798)		5	1			
	^2^Pterostichus (Platysma) niger (Schaller, 1783)	1	2				
	^2^Pterostichus (Pseudomaseus) minor (Gyllenhal, 1827)						1
Cerambycidae	^1^*Alosternatabacicolor* (De Geer, 1775)		1			8	2
	^1^*Leiopuslinnei* Wallin, Nylander & Kvamme, 2009						4
	^1^*Leiopusnebulosus* (Linnaeus, 1758)		1	4			1
	^1^*Lepturaquadrifasciata* Linnaeus, 1758		1				1
	^1^*Pogonocherushispidus* (Linnaeus, 1758)					1	
	^1^*Pyrrhidiumsanguineum* (Linnaeus, 1758)						8
	^1^Rhagium (Megarhagium) mordax (De Geer, 1775)	9	16	7	13	11	20
	^1^Rhagium (Rhagium) inquisitor Linnaeus, 1758	1					
	^1^*Saperdaperforata* (Pallas, 1773)		1				
	^1^*Saperdascalaris* (Linnaeus, 1758)			4	1		3
	^1^*Stictolepturarubra* (Linnaeus, 1758)		1				
	^1^*Xylotrechusrusticus* (Linnaeus, 1758)				1		
Cerylonidae	^1^*Cerylondeplanatum* Gyllenhal, 1827		3	2	1	2	6
	^1^*Cerylonfagi* Brisout de Barneville, 1867	1		2	2	1	1
	^1^*Cerylonferrugineum* Stephens, 1830	1			6		1
	^1^*Cerylonhisteroides* (Fabricius, 1792)	1	1	2			2
Chrysomelidae	^3^*Batophilarubi* (Paykull, 1799)		1				
	^3^Cassida (Cassida) nebulosa Linnaeus, 1758	1					
	^3^*Chaetocnemahortensis* (Geoffroy, 1785)	1	1				
	^3^*Chaetocnemapicipes* Stephens, 1831			1			
	^3^Cryptocephalus (Burlinius) rufipes (Goeze, 1777)	1					
	^3^Galeruca (Galeruca) tanaceti (Linnaeus, 1758)		1				
	^3^*Longitarsusmelanocephalus* (De Geer, 1775)				1	1	
	^3^*Lythrariasalicariae* (Paykull, 1800)					1	
	^3^*Oulemamelanopus* (Linnaeus, 1758)			1	1		
	^3^Phaedon (Phaedon) cochleariae (Fabricius, 1792)	1	1				
	^3^*Phyllobroticaquadrimaculata* (Linnaeus, 1758)				1		
	^3^*Phyllotretaatra* (Fabricius, 1775)		2		1		
	^3^*Phyllotretanemorum* (Linnaeus, 1758)			1			
	^3^*Phyllotretastriolata* (Fabricius, 1803)	1	1				
	^3^*Phyllotretaundulata* Kutschera, 1860	1				1	
	^3^*Phyllotretavittula* (Redtenbacher, 1849)	1	7	1		1	1
	^3^Psylliodes (Psylliodes) napi (Fabricius, 1792)						3
Ciidae	^1^*Cisalter* Silfverberg, 1991				1		
	^1^*Cisglabratus* Mellié, 1848						1
	^1^*Cisjacquemartii* Mellié, 1848				4		2
	^1^*Cisboleti* (Scopoli, 1763)				3		
	^1^*Ciscastaneus* Mellie, 1848					1	
	^1^*Cismicans* (Fabricius, 1792)	1				1	
	^1^*Ennearthroncornutum* (Gyllenhal, 1827		1				
	^1^*Orthocisfestivus* (Panzer, 1793)				1		
Cleridae	^1^*Thanasimusformicarius* (Linnaeus, 1758)	7		2	1		4
Coccinelidae	^3^*Calviadecemguttata* (Linnaeus, 1758)				1		
	^3^*Chilocorusrenipustulatus* (Scriba, 1790)		1				
	^3^*Propyleaquatuordecimpunctata* (Linnaeus, 1758)	1	1			1	1
	^3^Scymnus (Pullus) suturalis Thunberg, 1795			1			
	^3^*Tytthaspissedecimpunctata* (Linnaeus, 1758)						1
Corylophidae	^1^*Orthoperuspunctatus* (Wankowicz, 1865)						2
	^1^*Orthoperusatomus* (Gyllenhal, 1808)	1		1	2	1	8
	^1^*Orthoperusrogeri* Kraatz, 1874			2			
	^2^*Sericoderuslateralis* (Gyllenhal, 1827)		1	1	3		
Cryptophagidae	^1^*Atomariaprocerula* Erichson, 1846	1					
	^7^*Atomariapusilla* (Paykull, 1798)						1
	^1^Atomaria (Agathengis) nigrirostris Stephens, 1830	2	4	1			2
	^7^Atomaria (Atomaria) fuscipes (Gyllenhal, 1808)	1					
	^1^Atomaria (Atomaria) turgida Erichson, 1846			1			
	*Atomaria* sp. Stephens, 1829	1	1	2	1		2
	^1^*Caenoscelisferruginea* (Sahlberg, 1820)			1			1
	^1^*Caenoscelissubdeplanata* Brisout de Barneville, 1882			1			
	^1^*Cryptophagusdorsalis* Sahlberg, 1819		1				
	^1^*Cryptophagusbadius* Sturm, 1845	3			2		
	^1^*Cryptophagusdentatus* (Herbst, 1793)			1	1	2	
	^1^*Cryptophagusfuscicornis* Sturm, 1845	1					
	*Cryptophagus* sp. Herbst, 1792	2	1			1	
	^1^*Cryptophaguspallidus* Sturm, 1845	4					1
	^1^*Cryptophaguspilosus* Gyllenhal, 1827	5	1		5		5
	^2^*Cryptophagussetulosus* Sturm, 1845		1				
	^2^*Ephistemusglobulus* (Paykull, 1798)		4		19		10
	^1^*Ephistemusreitteri* Casey, 1900		1				
	^1^*Micrambeabietis* (Paykull, 1798)	4		5			
Cucujidae	^1^*Cucujuscinnaberinus* (Scopoli, 1763)		13		2	1	5
Curculionidae	^1^Acalles (Acalles) camelus (Fabricius, 1792)				1		
	^3^*Brachysomusechinatus* (Bonsdorff, 1785)		5		3	2	5
	^3^*Ceutorhynchusnapi* Gyllenhal, 1837		1				
	^3^*Ceutorhynchuspallidactylus* (Marsham, 1802)						2
	^1^*Crypturguscinereus* (Herbst, 1793)		4				
	^1^*Crypturgushispidulus* Thomson, 1870		1			3	
	^1^*Crypturguspusillus* (Gyllenhal, 1813)				34	2	21
	^3^Curculio (Curculio) glandium Marsham, 1802						1
	^3^Curculio (Curculio) nucum Linnaeus, 1758						1
	^1^*Dryocoetesalni* (Georg, 1856)		6	1		1	
	^1^*Dryocoetesautographus* (Ratzeburg, 1837)		217	3		2	1
	^1^*Dryocoetesvillosus* (Fabricius, 1792)						1
	^1^*Ernoporustiliae* (Panzer, 1793)						1
	^1^*Hylastesater* (Paykull, 1800)		1		2	1	
	^1^*Hylesinuscrenatus* (Fabricius, 1787)	94				59	59
	^1^Hylobius (Callirus) abietis (Linnaeus, 1758)				3		
	^3^Hypera (Hypera) postica (Gyllenhal, 1813)				1		
	^3^Otiorhynchus (Choilisanus) raucus (Fabricius, 1777)		7				
	^3^Otiorhynchus (Nihus) scaber (Linnaeus, 1758)	2		1			
	^3^Phyllobius (Dieletus) argentatus (Linnaeus, 1758)				3	12	2
	^3^Phyllobius (Metaphyllobius) glaucus (Scopoli, 1763)					2	
	^1^*Pityogeneschalcographus* (Linnaeus, 1761)	2					
	^3^Polydrusus (Eustolus) corruscus Germar, 1824		1				
	^3^*Sciaphilusasperatus* (Bonsdorff, 1785)	1			3	27	7
	^1^*Scolytusratzeburgii* Janson, 1856				10		
	^3^*Stereonychusfraxini* (De Geer, 1775)					1	
	^3^Strophosoma (Strophosoma) capitatum (De Geer, 1775)		5		28	14	28
	^1^*Taphrorychusbicolor* (Herbst, 1793)	2	23			1	
	^1^Trachodes (Trachodes) hispidus (Linnaeus, 1758)		3	1	2	1	1
	^1^*Trypodendrondomesticum* (Linnaeus, 1758)		2	7			
	^1^*Trypodendronlineatum* (Olivier, 1795)		2	45	15		
	^1^*Trypodendronsignatum* (Fabricius, 1787)	77	16	718	401	6	7
	^1^*Xyleborinussaxesenii* (Ratzeburg, 1837)					1	2
	^1^*Xyleboruscryptographus* (Ratzeburg, 1837)		62				
	^1^*Xyleborusdispar* (Fabricius, 1792)	77	166	54	1	6	9
Dasytidae	^1^Dasytes (Dasytes) niger (Linnaeus, 1761)					1	
	^1^Dasytes (Mesodasytes) plumbeus (Muller, 1776)					1	
Dermestidae	^5^Anthrenus (Florilinus) museorum (Linnaeus, 1761)						1
	^6^Dermestes (Dermestes) ater De Geer, 1774					1	
	^5^Dermestes (Dermestinus) murinus Linnaeus, 1758					1	
Elateridae	^1^Ampedus (Ampedus) erythrogonus (Muller, 1821)				1		1
	^1^Ampedus (Ampedus) nigrinus (Herbst, 1784)	4	3		1		1
	^1^Ampedus (Ampedus) pomorum (Herbst, 1784)	5	5		9		3
	^1^*Anostiruscastaneus* (Linnaeus, 1758)					1	1
	^1^Athous (Athous) haemorrhoidalis (Fabricius, 1801)	1					
	^2^Athous (Athous) vittatus (Gmelin, 1790)						1
	^1^Athous (Haplathous) subfuscus (Muller, 1764)	3		1			
	^1^*Dalopiusmarginatus* (Linnaeus, 1758)	6	7	4	2	7	11
	^1^*Denticollislinearis* (Linnaeus, 1758)	3	1	3	3	3	3
	^1^*Denticollisrubens* Piller & Mitterpacher, 1783		1				
	^1^*Diacanthousundulatus* (De Geer, 1774)		2	2		2	6
	^3^*Ectinusaterrimus* (Linnaeus, 1761)				1	1	
	^2^*Hemicrepidiusniger* (Linnaeus, 1758)					1	
	^1^Melanotus (Melanotus) castanipes (Paykull, 1800)	1	2		2	1	1
	^1^Melanotus (Melanotus) villosus (Fourcroy, 1785)			1		1	1
	^2^Selatosomus (Pristilophus) cruciatus (Linnaeus, 1758)						2
Endomychidae	^1^*Endomychuscoccineus* (Linnaeus, 1758)	3	10	4	14	3	3
	^1^*Leiestesseminiger* (Gyllenhall, 1808)						1
	^1^*Mycetinacruciata* (Schaller, 1783)				2	1	6
Erotylidae	^1^Dacne (Dacne) bipustulata (Thunberg, 1781)						1
	^1^*Triplaxrussica* (Linnaeus, 1758)	1					3
Eucnemidae	^1^*Eucnemiscapucina* Ahrens, 1812				1		
	^1^*Hylisprocerulus* (Mannerheim, 1823)		1		2	1	1
	^1^*Isorhipismelasoides* (Laporte de Castelnau, 1835)		1				
	^1^*Microrhagusemyi* (Rouget, 1856)	1					
	^1^*Microrhaguslepidus* Rosenhauer, 1847	1			1	1	1
	^1^*Microrhaguspygmaeus* (Fabricius, 1792)					1	
	^1^*Xylophilustestaceus* (Herbst, 1806)		1				
Geotrupidae	^2^*Anoplotrupesstercorosus* (Scriba, 1791)	3	19	1	7	2	
Histeridae	^1^Paromalus (Paromalus) parallelepipedus (Herbst, 1792)						1
Hydraenidae	^2^Hydraena (Hydraena) britteni Joy, 1907			1			
Kateretidae	^3^*Brachypterusglaber* (Newman, 1834)	1					
	^3^*Brachypterusurticae* (Fabricius, 1792)						2
Laemophloeidae	^1^*Cryptolestesferrugineus* (Stephens, 1831)				1		
	^1^*Placonotustestaceus* (Fabricius, 1787)	1	1				
Lampyridae	^2^*Phosphaenushemipterus* (Goeze, 1777)			1			
Latridiidae	^1^Cartodere (Aridius) nodifer (Westwood, 1839)	1	11	3	4	3	2
	^2^*Corticariaferruginea* Marsham, 1802		1				2
	^1^*Corticariafulva* (Comolli, 1837)		1				
	^1^*Corticarialongicollis* (Zetterstedt, 1838)			1	1		
	^2^*Corticarialongicornis* (Herbst, 1783)				2		
	^1^*Corticariaserrata* (Paykull, 1798)						1
	^1^*Corticarinaminuta* (Fabricius, 1792)	6	8		2		1
	*Corticarina* sp. Reitter, 1880	1					
	^1^*Corticarinasimilata* (Gyllenhal, 1827)			2	14	1	12
	^2^*Corticarinatruncatella* (Mannerheim, 1844)				1		
	^1^*Cortinicaragibbosa* (Herbst, 1793)	18	7	4	7	25	37
	^1^*Enicmusfungicola* Thomson, 1868		2		2	1	2
	^1^*Enicmusrugosus* (Herbst, 1793)	2	1		9	1	12
	^1^*Enicmustestaceus* (Stephens, 1830)				10		6
	^1^*Enicmustransversus* (Olivier, 1790)		1		1	1	
	^2^*Latridiusassimilis* (Mannerheim, 1844)				1		
	^1^*Latridiusconsimilis* (Mannerheim, 1844)				3	1	
	^1^*Latridiushirtus* (Gyllenhal, 1827)		7		3	2	2
	^1^*Latridiusminutus* (Linnaeus, 1767)	3	9	12	4	3	10
	^2^*Latridiusporcatus* Herbst, 1793				1		
	^2^Melanophthalma (Melanophthalma) transversalis (Gyllenhal, 1827)		1				
	^1^*Stephostethusangusticollis* (Gyllenhal, 1827)	4		1	3		
	*Stephostethus* sp. LeConte, 1878			1			
	^1^*Stephostethuspandellei* (Brisout, 1863)	3	4		2	2	1
	^1^*Stephostethusrugicollis* (Olivier, 1790)	1	1	1			
	^2^*Thesbergrothi* (Reitter, 1880)					1	
Leiodidae	^1^*Agathidiumatrum* (Paykull, 1798)						1
	^8^*Agathidiumlaevigatum* Erichson, 1845					4	
	^1^Agathidium (Agathidium) pisanum Brisout, 1872		2		3	1	1
	^1^Agathidium (Agathidium) seminulum (Linnaeus, 1758)			2	2	2	1
	^1^Agathidium (Neoceble) confusum Brisout, 1863	1	1			1	
	^8^Agathidium (Neoceble) convexum Sharp, 1866				1		
	^1^Agathidium (Neoceble) nigripenne (Fabricius, 1792)	1	38	6	1		1
	^8^Agathidium (Neoceble) rotundatum (Gyllenhal, 1827)		1		1		1
	^8^Agathidium (Neoceble) varians Beck, 1817		3				1
	^1^*Anisotomacastanea* (Herbst, 1792)		1	1		2	
	^1^*Anisotomaglabra* (Fabricius, 1792)	1	3				2
	^1^*Anisotomahumeralis* (Fabricius, 1792)	1	1	1	4	2	11
	^1^*Anisotomaorbicularis* (Herbst, 1792)	1	4	2	2	5	3
	^6^*Catopsnigrita* Erichson, 1837		4	1	4	3	
	*Catops* sp. Paykull, 1798	1	3				
	^4^*Colenisimmunda* (Sturm, 1807)						1
	^7^Colon (Myloechus) brunneum (Latreille, 1807)						1
	^7^*Colonviennense* Herbst, 1797					1	
	^8^*Fissocatopswesti* (Krogerus, 1931)			3	1		
	^8^*Hydnobiusspinipes* (Gyllenhal, 1813)		1				
	^8^*Leiodespallens* (Sturm, 1807)					1	
	*Leiodes* sp. Latreille, 1796					1	
	^1^*Liodopriaserricornis* (Gyllenhal, 1813)				1		
	^8^Nargus (Nargus) velox (Spence, 1815)		1				
	*Nargus* sp. Thomson, 1867			1			
	^8^Ptomaphagus (Ptomaphagus) varicornis (Rosenhauer, 1847)			1		1	
	^8^*Sciodrepoideswatsoni* (Spence, 1815)	1	2	6		6	
Lucanidae	^1^*Dorcusparallelipipedus* (Linnaeus, 1785)						1
	^1^*Sinodendroncylindricum* (Linnaeus, 1758)					1	
Lycidae	^1^*Lygistopterussanguineus* (Linnaeus, 1758)						3
Lymexylidae	^1^*Hylecoetusdermestoides* (Linnaeus, 1861)			38	52	5	2
	^1^*Lymexylonnavale* (Linnaeus, 1758)						1
Malachiidae	^1^*Malachiusbipustulatus* (Linnaeus, 1758)					1	2
Melandryidae	^1^*Hypulusquercinus* (Quensel, 1790)						5
	^1^*Melandryadubia* (Schaller, 1783)	1	2		2	1	3
	^1^Orchesia (Clinocara) undulata Kraatz, 1853	7	8	8	4	2	8
	^1^Orchesia (Orchesia) micans (Panzer, 1794)		1		1		2
Melolonthidae	^1^*Sericabrunnea* (Linnaeus, 1758)		1	3	3	3	4
Monotomidae	^3^Monotoma (Monotoma) picipes Herbst, 1793		1				
	^1^*Rhizophagusfenestralis* (Linnaeus, 1758)	10	20	3	8	13	5
	^1^Rhizophagus (Anomophagus) puncticollis Sahlberg, 1837	1					
	^1^Rhizophagus (Cyanostolus) aeneus Richter, 1820						2
	^1^Rhizophagus (Rhizophagus) bipustulatus (Fabricius, 1792)	9	3	5	4	4	4
	^1^Rhizophagus (Rhizophagus) dispar (Paykull, 1800)	11	3	30		5	1
	^1^Rhizophagus (Rhizophagus) ferrugineus (Paykull, 1800)			1			
	^1^Rhizophagus (Rhizophagus) nitidulus (Fabricius, 1798)	1	1	3			
	^1^Rhizophagus (Rhizophagus) oblongicollis Blatch & Horner, 1892			2			
Mordellidae	^1^Mordellistena (Mordellistena) humeralis (Linnaeus, 1758)				1		
	^1^*Tomoxiabucephala* (Costa, 1854)		2		1		1
Mycetophagidae	^1^Litargus (Litargus) connexus (Geoffroy, 1785)				1		3
	^1^Mycetophagus (Mycetophagus) quadripustulatus (Linnaeus, 1761)				1		
	^1^Mycetophagus (Mycetoxides) fulvicollis Fabricius, 1793						1
	^1^Mycetophagus (Philomyces) populi Fabricius, 1798				1		
	^1^Mycetophagus (Ulolendus) atomarius (Fabricius, 1787)						3
	^1^Mycetophagus (Ulolendus) piceus (Fabricius, 1777)						1
Nemonychidae	^3^*Cimberisattelaboides* (Fabricius, 1787)			1			
Nitidulidae	^3^*Brassicogethesaeneus* (Fabricius, 1775)	1	1			1	1
	^3^*Brassicogethessubaeneus* (Sturm, 1845)	1	1				
	^2^*Carpophilusligneus* Murray, 1864						1
	^1^*Cychramusluteus* (Fabricius, 1787)	3	2	10	3	38	5
	^1^*Cychramusvariegatus* (Herbst, 1792)	4	3	3	4	7	3
	^1^*Cyllodesater* (Herbst, 1792)						1
	^1^*Epuraeaangustula* Sturm, 1844	1		1			
	^1^*Epuraeamarseuli* Reitter, 1872	10		11	1		
	^2^*Epuraeamelanocephala* (Marsham, 1802)					1	
	^1^*Epuraeaneglecta* (Heer, 1841)	6	21	10	5		4
	^1^*Epuraeaoblonga* (Herbst, 1793)			1			
	^1^*Epuraeapallescens* (Stephens, 1835)	3	3	3	2		
	^1^*Epuraeaunicolor* (Olivier, 1790)	10		2			
	^1^*Epuraeavariegata* (Herbst, 1793)	8	7	3	8		1
	^1^*Glischrochilushortensis* (Geoffroy in Fourcroy, 1785)	93	51	11	6	1	6
	^1^*Glischrochilusquadriguttatus* (Fabricius, 1776)	2	21		2		14
	^1^*Glischrochilusquadripunctatus* (Linnaeus, 1758)		1				
	^1^*Glischrochilusquadrisignatus* (Say, 1835)				2		
	^1^*Ipidiabinotata* Reitter, 1875			1	2		2
	^3^*Lamiogethesbrunnicornis* (Sturm, 1845)						1
	^1^*Soroniapunctatissima* (Illiger, 1794)	1		1			
Phalacridae	^3^*Olibrusaffinis* (Sturm, 1807)						3
	^3^*Olibrusaeneus* (Fabricius, 1792)					1	
	^3^*Stilbustestaceus* (Panzer, 1797)					1	
	^3^*Stilbusoblongus* (Erichson, 1845)		4				
Ptiliidae	^2^Acrotrichis (Acrotrichis) sitkaensis (Motschulsky, 1845)			3			
	*Acrotrichis* sp. Motschulsky, 1848	1	11	8			
	^1^*Baeocraravariolosa* (Mulsant & Rey, 1873)	9	4	1			
	^2^*Nephanestitan* (Newman, 1834)	1					
	^1^Ptenidium (Gillmeisterium) nitidum (Heer, 1841)			1			1
	^1^Ptenidium (Matthewsium) turgidum Thomson, 1855		1				
	^1^Ptenidium (Ptenidium) pusillum (Gyllenhal, 1808)			1			2
	^1^*Ptinellaaptera* (Guérin-Méneville, 1839)	14	1				
	^1^*Ptinellalimbata* (Heer, 1841)	5	3		1		1
Pyrochroidae	^1^*Pyrochroacoccinea* (Linnaeus, 1761)	3	1		5		9
	^1^*Schizotuspectinicornis* (Linnaeus, 1758)	5	1		2	8	10
Salpingidae	^1^*Salpingusplanirostris* (Fabricius, 1787)				1	1	1
	^1^*Salpingusruficollis* (Linnaeus, 1761)	13	2	11	2	2	2
	^1^*Vincenzellusruficollis* (Panzer, 1794)					1	
Scirtidae	^2^*Cyphonochraceus* Stephens, 1830	18	16	306	66	2	
	^2^*Cyphonpadi* (Linnaeus, 1758)		2		2		1
	^2^*Microcaratestacea* (Linnaeus, 1767)	1		13	4		
Scraptiidae	^1^*Anaspisbrunnipes* (Mulsant, 1856)						1
	^1^Anaspis (Anaspis) frontalis (Linnaeus, 1758)		3	1	2	1	2
	^1^Anaspis (Anaspis) thoracica (Linnaeus, 1758)	7	2	2	2	2	17
	^1^*Scraptiafuscula* Muller, 1821						1
Scydmaenidae	^1^Microscydmus (Microscydmus) nanus (Schaum, 1844)						1
	^1^*Neurapheselongatulus* (Müller & Kunze, 1822)				1		
	^1^Scydmaenus (Parallomicrus) rufus Muller & Kunze, 1822	1					
	^1^*Scydmoraphesminutus* (Chaudoir, 1845)						1
	^1^Stenichnus (Cyrtoscydmus) collaris (Muller & Kunze, 1822)				1	2	
	^1^Stenichnus (Cyrtoscydmus) godarti (Latreille, 1806)				1	3	6
	^1^Stenichnus (Cyrtoscydmus) scutellaris (Muller & Kunze, 1822)			1	2		1
Silphidae	^6^*Nicrophorusvespillo* (Linnaeus, 1758)				1		
	^6^*Nicrophorusvespilloides* Herbst, 1783	1	5		2		
	^6^*Oiceoptomathoracicum* (Linnaeus, 1758)	1					
	^6^*Phosphugaatrata* (Linnaeus, 1758)	1	4	1	1	2	2
	^6^*Silphacarinata* Herbst, 1783					2	2
Silvanidae	^1^*Dendrophaguscrenatus* (Paykull, 1799)		2			1	
	^1^*Silvanoprusfagi* (Guérin-Méneville, 1844)		1				1
	^1^*Silvanusbidentatus* (Fabricius, 1792)		1				
	^1^*Silvanusunidentatus* (Olivier, 1790)	1					
Sphindidae	^1^*Aspidiphorusorbiculatus* (Gyllenhal, 1808)	5	98	4	50	35	12
Staphylinidae	^2^*Acrotonasylvicola* (Kraatz, 1856)		5				
	^1^*Acruliainflata* (Gyllenhal, 1813)	1		7			
	^2^*Aleocharaerythroptera* Gravenhorst, 1806			4			
	^7^*Aleocharafumata* Gravenhorst, 1802				1		
	^1^*Aleocharasparsa* Heer, 1839		14				
	^2^Aleochara (Euryodma) brevipennis Gravenhorst, 1806		1	2			
	^2^*Alevonotagracilenta* (Erichson, 1839)						1
	^2^*Aloconotagregaria* (Erichson, 1839)						4
	^5^*Amidobiatalpa* (Heer, 1841)				3		
	^2^*Amischaanalis* (Gravenhorst, 1802)		4				
	^1^*Anomognathuscuspidatus* (Erichson, 1839)				1		
	^2^*Anotylusrugosus* (Fabricius, 1775)		2				
	^2^*Athetaamicula* (Stephens, 1832)				3		
	^1^*Athetaatramentaria* (Gyllenhal, 1810)	3					
	^8^*Athetaboleticola* J. Sahlberg, 1876	10	5	12			
	^1^*Athetacrassicornis* (Fabricius, 1792)	3	4	55	23	5	
	^5^*Athetadivisa* (Märkel, 1844)	8	1				2
	^7^*Athetafungi* (Gravenhorst, 1806)	14	6	9	10	1	5
	^1^*Athetalaticollis* (Stephens, 1832)						2
	^2^*Athetalongicornis* (Gravenhorst, 1802)					3	
	^1^*Athetanigritula* (Gravenhorst, 1802)	8	7	19		1	
	*Atheta* sp. Thomson, 1858	41	7	2		4	5
	^1^Atheta (Atheta) hypnorum (Kiesenwetter, 1850)		1			8	3
	^1^*Atrecusaffinis* (Paykull, 1789)	2	5				
	^1^*Batrisodesdelaporti* (Aube, 1833)			1			
	^1^*Batrisodeshubenthali* Reitter, 1913						4
	*Batrisodes* sp. Reitter, 1882		1				
	^1^*Batrisodesvenustus* (Reichenbach, 1816)						1
	^1^*Bibloporusbicolor* (Denny, 1825)	3	4		2		1
	^2^*Bisniusfimetarius* (Gravenhorst, 1802)	3	1		2		1
	^2^*Bisniusnitidulus* (Gravenhorst, 1802)	1					
	^2^*Bolitobiuscastaneus* (Stephens, 1832)				1		
	^1^*Bolitocharalucida* (Gravenhorst, 1802)				1		2
	^1^*Bolitocharamulsanti* Sharp, 1875		2				
	^1^*Bolitocharaobliqua* Erichson, 1837	25	40	15	26	4	32
	^1^*Bolitocharapulchra* (Gravenhorst, 1806)		4				
	^2^*Brachyglutahaematica* (Reichenbach, 1816)				1		
	^1^*Carphacisstriatus* (Olivier, 1795)		1				
	^1^*Dadobiaimmersa* (Erichson, 1837)	2	1		1		
	^1^*Dinaraeaaequata* (Erichson, 1837)		7	5	1		3
	^1^*Dinaraeaangustula* (Gyllenhal, 1810)	4	1		2	4	
	^1^*Dinaraealinearis* (Gravenhorst, 1802)	4	10				4
	*Dinaraea* sp. Thomson, 1858		1				
	*Euplectus* sp. Leach, 1817	1	1				
	^1^*Euplectuspiceus* Motschulsky, 1835			1			5
	^1^*Euryusacastanoptera* Kraatz, 1856	2	3	4	9	2	2
	^2^Eusphalerum (Eusphalerum) minutum (Fabricius, 1792)			1	2		
	^2^*Gabriusbreviventer* (Sperk, 1835)	10		5		1	
	^2^*Gabriusnigritulus* (Gravenhorst, 1802)		1				
	^2^*Gabriusosseticus* (Kolenati, 1846)		4				
	^1^*Gabriussplendidulus* (Gravenhorst, 1802)	3	9	2	1		1
	^1^Geostiba (Geostiba) circellaris (Gravenhorst, 1806)		3			5	2
	^7^*Gyrophaenagentilis* Erichson, 1839				1		
	^7^*Gyrophaenajoyioides* Wüsthoff, 1937					2	
	^1^*Haploglossagentilis* (Märkel, 1844)					1	2
	^5^*Haploglossapulla* (Gyllenhal, 1827)					1	
	^1^*Homalotaplana* (Gyllenhal, 1810)				2	1	
	^8^*Hypnogyraangularis* (Ganglbauer, 1895)	1					
	^2^*Ilyobatesnigricollis* (Paykull, 1800)	16					
	^2^*Ischnosomasplendidum* (Gravenhorst, 1806)			2	1		
	^1^*Leptusapulchella* (Mannerheim, 1831)	13		7			
	^4^Lesteva (Lesteva) longoelytrata (Goeze, 1777)	1	2				
	^2^*Lioglutagranigera* (Kiesenwetter, 1850)			2			
	^1^*Lordithonpulchellus* (Mannerheim, 1830)					7	
	^1^*Lordithontrinotatus* (Erichson, 1839)					3	
	^1^*Lordithonlunulatus* (Linnaeus, 1760)	1	2		1		1
	^1^*Lordithontrimaculatus* (Fabricius, 1793)		1				
	^1^*Megarthrusdepressus* (Paykull, 1789)			1			
	^1^*Mycetoporuspunctus* (Gravenhorst, 1806)		1				
	^2^*Nehemitropialividipennis* (Mannerheim, 1831)			2	1		
	^1^*Nudobiuslentus* (Gravenhorst, 1806)		4		2	1	1
	^2^*Olophrumassimile* (Paykull, 1800)		1				
	^2^*Olophrumfuscum* (Gravenhorst, 1806)	1					
	^1^*Omaliumrivulare* (Paykull, 1789)	2					
	^1^*Othiuslapidicola* Markel & Kiesenwetter, 1848	1					
	^2^*Othiussubuliformis* Stephens, 1833		4			1	
	^3^*Oxypodaacuminata* (Stephens, 1832)	6	3			2	3
	^2^*Oxypodaannularis* (Mannerheim, 1830)				7		
	^3^*Oxypodabrevicornis* (Stephens, 1832)			24	1		5
	^2^*Oxypodaopaca* (Gravenhorst, 1802)		2		3	1	3
	^2^*Oxypodapraecox* Erichson, 1839				3	1	
	^1^Oxypoda (Mycetodrepa) alternans (Gravenhorst, 1802)	3	11	1	1		4
	*Oxypoda* sp. Mannerheim, 1831			4	1	2	2
	^2^*Philhygraelongatula* (Gravenhorst, 1802)	4	3		3	3	6
	^2^*Philhygraluridipennis* (Mannerheim, 1830)				1	1	
	*Philhygra* sp. Mulsant & Rey, 1873		5				1
	^2^*Philonthusdecorus* (Gravenhorst, 1802)					1	
	^1^Phloeonomus (Phloeonomodes) minimus (Erichson, 1839)						1
	^1^Phloeonomus (Phloeonomus) punctipennis Thomson, 1867	1		2		1	9
	^1^Phloeonomus (Phloeonomus) pusillus (Gravenhorst, 1806)					3	
	^1^*Phloeoporatestacea* (Mannerheim, 1830)	1			3		
	^1^*Phloeostibaplana* (Paykull, 1792)				5		8
	^1^Phyllodrepa (Dropephylla) ioptera (Stephens, 1832)						1
	^1^Phyllodrepa (Phyllodrepa) melanocephala (Fabricius, 1787)		1				
	^1^*Phyllodrepoideacrenata* (Gravenhorst, 1802)		1				
	^1^*Placusaincompleta* Sjöberg, 1934					1	
	^1^Placusa (Placusa) atrata (Mannerheim, 1831)	10	1	3			
	^1^Placusa (Placusa) tachyporoides (Waltl, 1838)	21	1				
	^1^*Plectophloeusfischeri* (Aube, 1833)		1	1	1	2	1
	^1^*Plectophloeusnubigena* Reitter, 1877						1
	^1^*Proteinusbrachypterus* (Fabricius, 1792)	4					
	^1^Quedius (Microsaurus) brevicornis (Thomson, 1860)		1				
	^1^Quedius (Microsaurus) cruentus (Olivier, 1795)		6	1	1		
	^5^Quedius (Microsaurus) longicornis Kraatz, 1857	2	2				
	^1^Quedius (Microsaurus) scitus (Gravenhorst, 1806)			1	1		
	^1^Quedius (Microsaurus) xanthopus Erichson, 1839	2	5		3		
	^1^Quedius (Quedionuchus) plagiatus Mannerheim, 1843		1				
	^4^Quedius (Quedius) fuliginosus (Gravenhorst, 1802)					6	
	^4^Quedius (Quedius) molochinus (Gravenhorst, 1806)	2					
	^1^*Saulcyellaschmidtii* (Maerkel, 1844)				1		
	^1^*Scaphidiumquadrimaculatum* Olivier, 1790				1		
	^1^*Scaphisomaagaricinum* (Linnaeus, 1758)	1	14		33	3	15
	^1^*Scaphisomaboleti* (Panzer, 1793)		1	3		2	
	^1^*Sepedophilusbipunctatus* (Gravenhorst, 1802)				1		
	^1^*Sepedophilusbipustulatus* (Gravenhorst, 1802)				15	1	22
	^1^*Sepedophiluslittoreus* (Linnaeus, 1758)	3	10	4	2		1
	^1^*Sepedophilustestaceus* (Fabricius, 1793)				2		
	^2^*Stenushumilis* Erichson, 1839				1		
	^2^*Stenusbimaculatus* Gyllenhal, 1810			1			
	^2^*Stenusjuno* (Paykull, 1789)	1	1				
	^2^*Stenuslustrator* Erichson, 1839	1					
	^2^*Syntomiumaeneum* (Muller, 1821)		2		1		2
	^2^*Tachinusfimetarius* Gravenhorst, 1802	2		1			
	^2^*Tachinuslaticollis* Gravenhorst, 1802			1			
	^2^*Tachinusmarginellus* (Fabricius, 1781)		3		1		
	^2^*Tachinussignatus* Gravenhorst, 1802	1					
	^2^*Tachyporushypnorum* (Fabricius, 1775)				1		2
	^4^*Tachyporusobtusus* (Linnaeus, 1767)					2	1
	^2^*Tachyporuspusillus* Gravenhorst, 1806						2
	^1^*Trimiumbrevicorne* (Reichenbach, 1816)					1	
	^1^*Tyrusmucronatus* (Panzer, 1805)						1
	^2^*Xantholinuslinearis* (Olivier, 1795)				1		
	^2^Xantholinus (Xantholinus) longiventris Heer, 1839				2		
	^2^*Xylodromusdepressus* (Gravenhorst, 1802)					2	
	^5^*Zyrashumeralis* (Gravenhorst, 1802)	3					
Tenebrionidae	^1^*Bolitophagusreticulatus* (Linnaeus, 1767)				19		
	^1^*Hypophloeusunicolor* (Piller & Mitterpacher, 1783)				11	3	
	^2^*Lagriahirta* (Linnaeus, 1758)		1				
	^1^*Ulomaculinaris* (Linnaeus, 1758)				1		
Tetratomidae	^1^*Hallomenusbinotatus* (Quensel, 1790)				1		
Throscidae	^2^*Trixaguscarinifrons* (Bonvouloir, 1859)			1		2	1
	^2^*Trixagusdermestoides* (Linnaeus, 1766)	9	15	11	92	12	60
Trogositidae	^1^*Nemozomaelongatum* (Linnaeus, 1761)			1			
Zopheridae	^1^*Colydiumelongatum* (Fabricius, 1787)						2
	^1^*Synchitahumeralis* (Fabricius, 1792)	2	1		3		1
		**Total**					
**Species**		162	205	143	198	159	210
**Individuals**		956	1383	1686	1347	556	859

^1^ Saproxylic species; ^2^ species related with accumulated organic matter, plant litter, mud and soil; ^3^ with various plants; ^4^ with mosses; ^5^ with nests of bird and other animals; ^6^ with carrion; ^7^ with fungal fruiting bodies; ^8^ habitat association unknown, but considered non-saproxylic.
